# Use of the Temperament and Character Inventory to describe the effectiveness of Gestalt therapy

**DOI:** 10.3389/fpsyt.2025.1280954

**Published:** 2025-09-26

**Authors:** Benjamin Calvet, Jean-Luc Vallejo, Yves Plu, Isabelle Soulat, Alexandra Foucher, Jean-Pierre Clément

**Affiliations:** ^1^ Institut National de la Santé Et de la Recherche Médicale (INSERM) U1094, Institut de Recherche pour le Développement (IRD) UMR270, Univ. Limoges, CHU Limoges, EpiMaCT - Epidemiology of chronic diseases in tropical zone, Institute of Epidemiology and Tropical Neurology, OmegaHealth, Limoges, France; ^2^ Pôle Universitaire de Psychiatrie de l’Adulte, de la Personne âgée et d’Addictologie, centre hospitalier Esquirol, Limoges, France; ^3^ Centre mémoire de ressources et de recherche du Limousin, centre hospitalier Esquirol, Limoges, France; ^4^ Institut Limousin de Formation et de Gestalt-Thérapie, Limoges, France; ^5^ Institut Gestalt+, Rennes, France

**Keywords:** Gestalt therapy, Temperament and Character Inventory, personality, effectiveness, temperament, character, anxiety, depression

## Abstract

**Background:**

Gestalt therapy (GT) is a dynamic, integrative, embodied approach that addresses human existence as a fundamentally relational modality, in which the experience of the self is situated in a dynamic organism–environment field. Assessments of GT are scarce, often avoided, and generally qualitative. The Therapy Gestalt TCI (THEGETCI) study aimed to show that the modifiable character dimensions of the 125-item Temperament and Character Inventory (TCI-125), as well as other personality temperament dimensions and emotional measures, change after a GT program.

**Methods:**

A psychotherapeutic program consisting of 33 one-hour sessions (minimum of 12 sessions), spaced over several months, was offered to 319 subjects with mood and/or anxiety disorders. The TCI-125, Hospital Anxiety and Depression Scale, and visual analog scale (VAS) were used to assess subjective psychological states before and after the program. All TCI-125 scores were adjusted for potential confounding factors.

**Results:**

Statistically significant differences between the initial and final mean scores were observed for anxiety (t = 16.46; p < 0.0001), depression (t = 11.24; p < 0.0001), and harm avoidance (t = 8.82; p < 0.0001), and global psychological distress assessed by VAS (t = 18.7; p < 0.0001) (all showing decreased scores). Significant increases were observed for the three maturity dimensions: Self-Directedness (t = −11.49; p < 0.0001), Cooperativeness (t = −2.77; p < 0.006), and Self-Transcendence (t = −4.52; p < 0.0001).

**Conclusion:**

The THEGETCI study is one of the first to demonstrate the effectiveness of specific, current GT strategies on personality dimensions using rigorous evaluation methods. Further research is needed to confirm these results and to better identify both the expected benefits for practitioners and the problem profiles most likely to benefit from GT.

## 1 Introduction

Gestalt therapy (GT) is a humanistic, experiential, integrative, and embodied approach that considers contact with one’s environment as the “simplest primary reality” for any living organism’s survival and growth (1). GT approaches human existence from a fundamentally relational modality, in which the experience of the self is situated in a dynamic organism–environment field that encompasses both the individual and their environment (1–6). The experience of contact (7) occurs in a space–time referred to as the contact boundary, where the relationship between the individual and their world takes shape (Gestalt) moment by moment. It is in this space–time, at the limit between the two—”here and now”—that the way a person functions, their self, can be observed, and psychological events unfold: “Our thoughts, our actions, our behaviors, our emotions, are different ways of experiencing the encounter with these border events (8)”. The self is defined as “the system of contacts, which always integrates perceptual and proprioceptive functions, motor and muscular functions, and organic needs”. It is the agent that responds to variations in dominant organic needs and the pressure of stimuli from the environment (1). The self is our specific way of being in the world at each moment and the agent of contact with the environment, which allows creative adjustment (9). GT focuses on the forms and disturbances, as well as inhibitions and interruptions, of the contact process that occurs during the session. GT is a relational therapy oriented toward processes (10). Gestalt therapists, through their attitude of presence, awareness, and responsibility, invite the patients to focus on the present—their thoughts, emotions, and movements—”here and now”. They help patients become aware of what is emerging in their current experience, rather than turning to specific, painful experiences from the past that have not been assimilated or anticipating a threatening or hostile future. Finally, they invite the clients to clarify their needs, goals, and values and to express them through role-playing or creative exercises (11). Presence, awareness, and responsibility are key characteristics of GT, which aims to restore the patients’ capacity for creative adjustment in their current environment and awaken new possibilities for growth and understanding (12). GT focuses on self-awareness, acceptance, personal responsibility, authenticity, and personal growth (13). Wholeness is a cornerstone of GT, which considers the field or context in which the client lives (14). Patients change only when they fully accept themselves and become who they are at present (15).

Studies on GT usually prefer narrative inquiries with data analysis involving the reduction, synthesis, and reconfiguration of clients’ stories (16). In their research, Kelly and Howie identified several emerging themes located in a temporal framework of experiences evolving through stages and as a circle (17). Cycles of experiences are micro- and macroscopic, and small and large, such as stories within stories (18). According to Clarkson (13), themes in the Gestalt experience cycle include fertile voids, sensation, awareness mobilization, action, contact, satisfaction, and withdrawal. A hallmark of GT is awareness of feelings, thoughts, actions, perceptions, and beliefs; awakening one’s awareness is its main purpose (19). Clients block or cloud their awareness through defects in projection, retroflection, confluence, and introjection. For therapists, the most important healing presence they can provide is caring, support, empathy, non-judgment, careful listening, seeing and hearing, a selective sense of humor, attention to meaning, and a lack of expectations about outcomes (20).

All exist in a unitary function, which is the total organism–environment process. Paying attention to any one of these components is appropriate for promoting personality integration (1). Even if GT presents the self as a function of three structures—namely, the id, ego, and personality (21)—which are the major stages of creative adjustment (7), assessing personality, particularly personality changes, is a way to consider progress in the psychotherapy process in outpatients with no major psychopathologies.

Assessments of GT are scarce, largely qualitative (22, 23), and often concerned with fidelity, adherence to therapists (24, 25), and aesthetic sensibility (2, 26–28). Only the Clinical Outcomes in Routine Evaluation–Outcome Measure (CORE-OM) has been used to measure clients’ levels of distress on a session-by-session basis, but it is more oriented toward comparing the effectiveness of counselors (29, 30).

Any psychotherapeutic process should be able to mobilize emotions, affect, and character; the Gestalt approach focuses on these objectives (31). This study aimed to assess the efficacy of GT and the usefulness of Cloninger’s Temperament and Character Inventory (TCI) as a robust tool for doing so. For Perls and Goodman, personality is a kind of “verbal copy of the self”: “[ … ] it is the hypothesis of what one is and which serves as a basis from which one would explain one’s own behavior if asked” (1). To adhere to this definition, we opted for a dimensional and lexical assessment method of personality and its evolution: the Cloninger’s TCI.

In 1987, Cloninger (32) postulated a unified biopsychosocial theory of personality that included four predominantly genetically determined temperamental dimensions and three predominantly developmentally determined character dimensions. Temperament dimensions are related to the immediate response of humans to basic environmental stimuli (danger, punishment, novelty, and reward). Character, by contrast, is described as a response to different aspects of one’s own identity or self-experience (individual differences in goal values and self-consciousness, and ego-syntonic emotions such as shame, guilt, and empathy). The aspects of the self correspond to how the self is identified as an autonomous individual, an integral part of society, and an integral part of all things (the universe). The three character dimensions are considered to be influenced by learning processes throughout the lifespan, including psychotherapeutic processes.

Within temperament, Novelty Seeking (NS) is thought to be related to the behavioral activation system (in response to novelty and signals of reward or relief from punishment). NS reflects the tendency to seek strong new sensations from unknown stimuli and reward signals. Harm Avoidance (HA) is related to the behavioral inhibition system (in response to punishment or non-reward signals) and corresponds to the tendency to respond to aversive stimuli with inhibition to avoid suffering, punishment, and frustration. Reward Dependence (RD) is related to the behavioral maintenance system (behaviors maintained without reinforcement) and is associated with being sentimental, warm, dedicated, and attached—versus withdrawn and cold. RD reflects the tendency to respond consistently to reward signals and to avoid punishment. The Persistence (P) dimension is related to perseverance—the ability to continue a behavior without considering its consequences in the absence of immediate reinforcement, despite fatigue and frustration.

Self-Directedness (SD) refers to individuals’ ability to control, regulate, and adapt their behavior in accordance with chosen goals and values. SD corresponds to individual maturity—the ability to adapt behavior based on values and personal orientations—which encompasses will to succeed, personal effectiveness, and self-esteem. Cooperativeness (C) relates to the tendency toward social tolerance, empathy, compassion, and helpfulness. C reflects social maturity, characterized by interpersonal relationships and social consciousness, as well as the acceptance of others. Self-Transcendence (ST) reflects spiritual maturity, designating spiritual realization and the ability for meditation and non-materialist thinking that allows the individual to represent the universe (33).

Cloninger et al. proposed several versions of the TCI (34). The version used in this study was a short form consisting of 125 self-reported true/false (yes/no) items (TCI-125). As in the original inventory, the TCI-125 temperament section includes 20 items for NS, 20 for HA, 15 for RD, and 5 for P; the character section includes 25 items for SD, 25 for C, and 15 for ST. Each dimension has one to five facets (see **Table 1**); thus, 25 facets were also measured as subscales of the seven main TCI scores.

**Table 1:** Mean comparisons (paired t-test) between initial and final TCI-125 facet scores with *post-hoc* Benjamini–Hochberg procedure to control false discovery rates.

	Initial mean (SD) n = 319	Final mean (SD) n = 319	*t*	P-value	B-Hp rank (*i*)	B-Hp critical B-H (*i*/*m* * *Q*)
Temperament						
Novelty Seeking						
Exploratory Excitability (NS1)	60.00 (29.37)	63.95 (27.02)	−2.64	0.009	17	0.034
Impulsiveness (NS2)	49.34 (31.59)	46.33 (30.61)	2.01	0.04^+^	18	0.036
Extravagance (NS3)	50.78 (26.90)	50.53 (23.83)	0.215	0.83^+^	25	0.05
Disorderliness (NS4)	37.12 (25.61)	37.43 (24.46)	−0.258	0.80^+^	24	0.048
Harm Avoidance						
Anticipatory Worry (HA1)	56.49 (30.10)	47.40 (30.23)	6.07	<0.0001	6	0.012
Fear of Uncertainty (HA2)	61.38 (31.81)	53.48 (31.92)	5.67	<0.0001	8	0.016
Shyness with Strangers (HA3)	58.43 (33.44)	47.33 (33.31)	7.13	<0.0001	4	0.008
Fatigability and Asthenia (HA4)	53.67 (32.10)	46.71 (32.76)	4.69	<0.0001	11	0.022
Reward Dependence						
Sentimentality (RD1)	78.43 (21.6)	75.80 (21.42)	2.762	0.006	15	0.03
Attachment (RD3)	64.26 (31.15)	72.23 (28.37)	−5.7	<0.0001	7	0.014
Dependence (RD4)	72.16 (24.26)	65.39 (24.34)	4.94	<0.0001	10	0.02
Persistence (P)	66.14 (29.63)	64.01 (28.84)	1.98	0.114^+^	20	0.04
Character						
Self-Directedness						
Responsibility (SD1)	58.93 (30.55)	73.73 (27.35)	−9.09	<0.0001	1	0.002
Purposefulness (SD2)	53.42 (28.95)	67.90 (28.07)	−8.46	<0.0001	2	0.004
Resourcefulness (SD3)	61.38 (29.85)	72.23 (27.65)	−7.55	<0.0001	3	0.006
Self-Acceptance (SD4)	64.26 (30.58)	72.16 (29.93)	−5.22	<0.0001	9	0.018
Congruent Second Nature (SD5)	61.94 (28.15)	71.28 (25.78)	−6.39	<0.0001	5	0.01
Cooperativeness						
Social Acceptance (C1)	80.06 (24.49)	83.39 (22.51)	−3.12	0.002	13	0.026
Empathy (C2)	79.25 (22.75)	80.38 (21.45)	−0.97	0.331^+^	21	0.042
Helpfulness (C3)	78,75 (15.49)	79.69 (15.00)	−0.96	0.339^+^	22	0.044
Compassion (C4)	86.08 (22.64)	87.84 (20.56)	−1.81	0.072^+^	19	0.038
Integrated Conscience (C5)	88.90 (13.31)	88.59 (14.72)	0.37	0.716^+^	23	0.046
Self-Transcendence						
Self-Forgetfulness (ST1)	40.88 (31.71)	44.20 (31.94)	−2.66	0.008	16	0.032
Transpersonal Identification (ST2)	50.66 (28.71)	56.11 (28.37)	−3.97	<0.0001	12	0.024
Spiritual Acceptance (ST3)	41.69 (24.84)	45.39 (25.25)	−3.07	0.002	14	0.028

Negative *t*-values indicate an increase in post-treatment scores due to subtraction order (initial − final). All TCI-125 scores were controlled for age, sex, socioeconomic condition, medication use, duration of therapy, and anxiety and depression scores using analysis of covariance (ANCOVA).

B-Hp, Benjamini–Hochberg procedure with *m* = 25 and *Q* = 5%.

^+^Non-significant after Benjamini–Hochberg adjustment.

Each facet corresponds to two opposing poles within its respective dimension, enabling the assessment of higher or lower dimensional levels depending on proximity to either pole. For NS, high Exploratory Excitability (NS1) indicates enjoyment of unfamiliar situations and a tendency to seek novelty, thrills, and adventure. High Impulsiveness (NS2) suggests poor impulse control and hasty decision-making. High Extravagance (NS3) is linked to excessive spending of money, energy, or emotions. High Disorderliness (NS4) is associated with irritability and disorganization. In HA, high Anticipatory Worry (HA1) indicates anxiety or pessimism, often expecting danger or failure in relatively safe situations. High Fear of Uncertainty (HA2) reflects discomfort with ambiguity and unfamiliar circumstances perceived as threatening. High Shyness with Strangers (HA3) indicates low self-confidence in social settings, whereas high Fatigability and Asthenia (HA4) is linked to low energy and susceptibility to exhaustion. In RD, high Sentimentality (RD1) indicates emotional sensitivity and compassion. High Attachment (RD3) reflects a preference for sharing experiences and forming warm, lasting bonds. High Dependence (RD4) indicates reliance on others for emotional support and approval. The P dimension has no subfacets; high scores indicate perseverance, resourcefulness, and resilience despite frustration and fatigue. In SD, high Responsibility (SD1) reflects autonomy and accountability; high Purposefulness (SD2) indicates goal orientation and determination; high Resourcefulness (SD3) reflects competence and problem-solving ability; high Self-Acceptance (SD4) indicates confidence and acceptance of strengths and limitations; and high Congruent Second Nature (SD5) reflects habitual alignment of actions with values and long-term goals. In C, high Social Acceptance (C1) reflects tolerance and friendliness; high Empathy (C2) reflects perspective-taking; high Helpfulness (C3) is associated with supportive, encouraging, or reassuring behaviors; high Compassion (C4) reflects kindness, forgiveness, and generosity; and high Integrated Conscience (C5) reflects fairness, sincerity, and principled behavior. In ST, high Creative Self-Forgetfulness (ST1) reflects the ability to transcend personal boundaries during deep engagement; high Transpersonal Identification (ST2) reflects a profound connection with nature and the universe; and high Spiritual Acceptance (ST3) indicates belief in miracles or supernatural experiences, sometimes involving magical thinking. The main objective of this study was to show that modifiable character dimensions, other personality traits (temperament), and emotional measures change as a result of GT. The secondary objective was to demonstrate that these changes are specifically related to motives for seeking psychotherapeutic help and to the specific psychotherapeutic alternatives that GT can offer for different types of suffering or disorders (see **Table 2**) that generate these motives. Therefore, the results for each type are discussed separately.

**Table 2:** Therapeutic strategies currently proposed in Gestalt therapy.

Character dimensions and facets with significant score increase		Problems and proposed strategies
	INC, p	
		Neurotic problems (n = 136)
Self-Directedness	****	Increase confidence in oneself and in one’s life goals by revisiting past negative experiences and confronting them with the reality of the present. Work on the ability to make choices based on one’s own resources and values rather than those of others (addressing introjects)
SD1 Responsibility	****	Become aware of repetitive and ineffective feelings and behaviors. Stimulate freedom of choice and experimentation with alternative, beneficial, and self-determined solutions aligned with life goals. Work on accepting one’s potential and limits and exploring the different polarities and facets of personality.
SD2 Purposefulness	****	
SD3 Resourcefulness	****	
SD4 Self-Acceptance	**	
SD5 Congruent Second Nature	***	Work on the “then”—implementing therapeutic gains into daily life.
C1 Social Acceptance C4 Compassion	* *	Address projections onto the “other” and accept one’s own motivations and interests. Support compassion for self and others, reducing desires for reparation or revenge.
Self-Transcendence	****	Practice meditation and global focusing (breathing, sensations, images, emotions, movements, and thoughts) on the current experience.
ST1 Self-Forgetfulness	*	Allow spiritual and/or hermeneutic experience to occur without falling into magical thinking. Enjoy the present moment without trying to control it. Let meaning emerge naturally. Stimulate creativity.
ST2 Transpersonal Identification	****	
ST3 Spiritual Acceptance	*	
		Emotional problems (n = 86)
Self-Directedness	****	Provide a safe framework free of negative emotions (fear, anger, shame, and emptiness) and painful sensations (sadness, anxiety, guilt, and feelings of failure). Focus on present moment bodily and relational awareness. Offer support and reassurance. Use creative media to channel emotions. Include body exercises (breathing). Validate positive emotions.
SD1 Responsibility	****	Encourage sharing and gradual expression of introjects, fears, doubts, inhibitions, and insecurity in a welcoming, predictable, and energizing environment. Teach emotional regulation via body exercises and creativity. Rediscover intimacy and warm connection in the here and now. Reduce dependency on others by recognizing that constant complaints or seeking reassurance may lead to criticism or rejection.
SD2 Purposefulness	****	
SD3 Resourcefulness	***	
SD4 Self-Acceptance	***	
SD5 Congruent Second Nature	****	Work on the “then”—integrating therapeutic benefits into daily life.
ST1 Self-Forgetfulness	*	Use meditation to seek meaning, stimulate spirituality, and foster detachment from suffering.
		Self-esteem problems (n = 41)
Self-Directedness	****	Strengthen self-confidence, trust in the therapist, and respect for authority. Improve responses to frustration and possible boundary transgressions in therapy.
SD1 *Responsibility*	****	Recognize repetitive, ineffective feelings and behaviors and their influence in therapy. Gradually address trauma affecting body, behaviors, and relationships. Support ongoing resources to build confidence and dynamism. Work on responsibility and forming balanced bonds—neither pejorated nor idealized—toward self and others.
SD3 *Resourcefulness*	**	
		Problems of loss or bereavement (n = 29)
Self-Directedness	****	Accept existential suffering, negative emotions (sadness, anger, shame, and emptiness), and painful sensations (anxiety, guilt, and feelings of rejection or helplessness). Focus on bodily and relational awareness in the present. Offer reassurance. Use role-playing to address absence.

Character dimensions and facets with significant score increase		Problems and proposed strategies
SD1 *Responsibility*	****	Introduce progressive meditation and global centering exercises (breathing, sensations, images, emotions, movements, and thoughts), respecting the patient’s pace. Encourage commitment to self-determined grief work (small rituals and writing) with positive effects. Stimulate untapped personal resources and strengthen beneficial family or friendship networks.
SD2 *Purposefulness*	****	
SD3 *Resourcefulness*	**	
SD4 *Self-Acceptance*	***	
SD5 *Congruent Second Nature*		Work on the “then”—applying therapeutic benefits to daily life.
ST3 *Spiritual Acceptance*	*	Work on accepting the inevitability of ruptures and death as part of life. Seek meaning—religious, philosophical, or human—in the face of loss.

Significance was determined using paired t-tests. The ANCOVA-adjusted results by group are presented in the Results and Discussion sections.

SD, Self-Directedness; C, Cooperativeness; ST, Self-Transcendence; INC, Increase.

****p ≤ 0.0001, ***p ≤ 0.001, ** p ≤ 0.01, and *p ≤ 0.05.

## 2 Materials and methods

### 2.1 Study design and participants

Therapy Gestalt TCI (THEGETCI) is an observational, longitudinal, prospective study. The investigators were Gestalt therapists informed of the goals (to promote the therapy), objectives (to show its efficacy), and methods. They were recruited through a network of students from the Limoges Institute of Formation in Gestalt Therapy and the Rennes Gestalt-plus Formation Center. All were graduates of accredited Gestalt institutes and certified by French and/or European professional bodies (FF2P, Fédération Française de Psychothérapie et de Psychanalyse; EAGT, European Association for Gestalt Therapy; and EAP, European Association for Psychotherapy). This study was approved by the local institutional review board (Ethics Committee of Limoges University Hospital, reference: CPP16-009/2015-A01208-41).

Although the therapists received payment from their clients, their participation in the study was voluntary and unrewarded. They were required to collect information about patients (age, sex, educational attainment, and usual medications) and briefly describe the reasons for clients’ requests for psychotherapy and its usefulness. The active client list of Gestalt psychotherapists is primarily composed of individuals with mild-to-moderate anxiety and depressive disorders (35, 36), often associated with neurotic, self-esteem, loss, grief, and separation issues. The authors examined the problems mentioned by patients, as well as the symptoms and behaviors identified by the psychotherapist, and classified them according to International Statistical Classification of Diseases and Related Health Problems 10th Revision into six categories: neurotic problems (F43.0 and F43.1), emotional problems (F32.0, F32.1, F41.0, F41.1, and F41.2), self-esteem problems (F60.30, F60.31, and F60.8), psychosomatic problems (F45.0 and F45.1), grief- and loss-related distress (F43.2), and other problems. These issues were examined separately to guide therapeutic strategies.

All therapists were trained by a group of experts in GT, and the content of care was formalized. All had been trained in GT institutes accredited by the European Association for GT and the European Association for Psychotherapy and were required to hold a European Certificate of Psychotherapy.

Following a semi-structured interview, therapists included clients meeting the inclusion and exclusion criteria and proposed a psychotherapeutic program consisting of 33 one-hour sessions (minimum of 12), spaced over several months, with a maximum duration of 2 years.

During the initial session, clinicians completed an observation note and asked patients to answer items related to personality, anxiety, and depression. Both therapist and patient independently assessed psychological distress using a 10-point visual analog scale.

The inclusion criteria were as follows: male or female patients aged 18 years or older, affiliated with health insurance, diagnosed with mood disorders (F32–F39, excluding bipolar affective disorders and manic episodes), possibly associated with an anxiety disorder (F41.0, F41.1, and F41.2) according to ICD-10 criteria, and with mild-to-moderate severity and no suicidal intent. All patients received oral and written explanations of the study’s purpose, research instruments, privacy protections, and their right to withdraw at any time. They provided written informed consent. The exclusion criteria were as follows: psychotic disorders, addictive behavior to one or more substances, cognitive deficits suggesting neurocognitive disorders, and legal protection status (guardianship).

There was no limit on the number of clients included during the 1-year recruitment period.

Of 59 Gestalt therapists (432 clients), 53 finalized 319 cases (77%). The main reasons for non-completion were abandonment (patient left before 12 sessions for personal, family, or work-related reasons) and loss to follow-up (patient left without explanation and was no longer in contact).

### 2.2 Measurement instruments and procedure

Individual development through psychotherapy was assessed using various tools. The primary instrument was the 125-item TCI, translated and validated in French (37, 38). Analyses were based on total scores for each dimension and facet (subdimension). All raw scores were weighted (out of 100) for readability.

Anxiety and depression were assessed using the validated French version of the Hospital Anxiety and Depression Scale (39, 40), a self-report questionnaire consisting of 14 four-point items: seven for anxiety and seven for depression, with each subscale scored from 0 to 21.

The visual analog scale (VAS) was used to assess subjective psychological states such as distress, anxiety, and moral pain. It is a simple, rapid, single-item measure in which the patients marked their perceived state on a visual line, and the therapists recorded their clinical global impression (41). The VAS is also suitable for assessing state changes. The scale consists of a line anchored by two extremes: “no distress” on the far left and “extreme distress” on the far right (42). Both patient and therapist marked the point corresponding to perceived intensity. Scores were calculated by measuring the distance in millimeters from the “no distress” anchor to the mark. VAS assessments were performed independently.

All measures were completed at the initial and final visits.

### 2.3 Statistical methods

Quantitative variables were described as means and standard deviations; qualitative variables were described as numbers and percentages. The Kolmogorov–Smirnov and Shapiro–Wilk tests were used to assess the normal distribution of quantitative variables. Paired t-tests were used to compare initial and final scores, given the one-to-one correspondence between samples (43), despite some deviations from normality, due to the test’s robustness with large samples (n > 30), as in this study (n = 319). All TCI scores were adjusted for potential confounders (age, sex, socioeconomic status, psychotropic medication use, duration of therapy, anxiety, and depression) using analysis of covariance (ANCOVA). All tests were two-sided, with the significance level set at α = 0.05 (p ≤ 0.05). To account for multiple comparisons, the Benjamini–Hochberg procedure was applied to control the false discovery rate, maintaining statistical power while limiting false positives (44). This procedure involves ranking p-values from multiple tests in ascending order and comparing each to a critical threshold calculated as (*i*/*m*) × α, where *i* is the rank, *m* is the total number of tests, and α is the predetermined false discovery rate (typically 0.05). A p-value is significant if ≤ (*i*/*m*) × α.

All analyses were performed using XLSTAT 2021.2.1 (45). and SPSS 29.0.2.0.

## 3 Results

The 319 subjects [78 men and 241 women; mean age = 41.01 ± 11.05 (range, 19–76)] had an average of approximately 31 ± 6 sessions (range, 12–53). The 53 therapists had an average of approximately six finalized cases each (range, 1–36). The mean duration between the initial and final sessions was 393 ± 150 days (range, 70–1155). Duration and number of sessions were statistically correlated (ρ = 0.36, p < 0.001). The evolution of different measures of mental state between the first and last sessions is presented in **Table 3** for the Hospital Anxiety and Depression Scale (HADS), TCI, and VAS assessment scales and in **Table 1** for the facets of the TCI. Post-treatment changes in the TCI dimensions remained significant in paired comparisons (**Table 3**) and were not explained by covariates such as age, sex, education level, psychotropic medication use, therapy duration, or baseline anxiety and depression scores. ANCOVA confirmed the independence of these changes from potential confounding variables, and these findings remained significant after applying the Benjamini–Hochberg procedure to control the false discovery rate.

**Table 3:** Mean comparisons (paired t-test) between initial and final scores with *post-hoc* Benjamini–Hochberg procedure to control false discovery rates.

	Initial mean (SD) n = 319	Final mean (SD) n = 319	*t*	P-value	B-Hp rank (*i*)	B-Hp critical B-H (*i*/*m* * *Q*)
HAD anxiety	10.87 (3.87)	7.58 (3.23)	16.46	<0.0001	3	0.0136
HAD depression	6.59 (3.99)	4,13 (2.98)	11.24	<0.0001	5	0.0227
TCI-125 Temperament:						
Novelty Seeking	49.31 (18.60)	49.56 (17.80)	−0.32	0.749^+^	11	0.05
Harm Avoidance	57.49 (24.84)	48.73 (25.66)	8.82	<0.0001	6	0.0273
Reward Dependence	71.62 (17.04)	71.14 (16.12)	0.62	0.533^+^	10	0.0454
Persistence	66.14 (29.63)	64.01 (28.84)	1.59	0.114^+^	9	0.0409
TCI-125 Character:						
Self-Directedness	59.99 (20.32)	71.46 (19.80)	−11.49	<0.0001	4	0.0189
Cooperativeness	82.61 (12.54)	83.97 (11.06)	−2.77	0.006	8	0.0364
Self-Transcendence	44.41 (23.84)	48.57 (24.19)	−4.52	<0.0001	7	0.0318

VAS patient	5.46 (2.18)	2.88 (2.04)	18.7	<0.0001	2	0.0091
VAS therapist	5.80 (1.98)	2.88 (2.03)	21.69	<0.0001	1	0.0045

Negative *t*-values indicate an increase in post-treatment scores due to subtraction order (initial − final). All TCI-125 scores were controlled for age, sex, socioeconomic condition, medication use, duration of therapy, and anxiety and depression scores using analysis of covariance (ANCOVA).

HAD, Hospital Anxiety and Depression Scale; TCI-125, 125-item Temperament and Character Inventory; VAS, visual analog scale for subjective psychological distress; B-Hp, Benjamini–Hochberg procedure with *m* = 11 and *Q* = 5%.

^+^Non-significant after Benjamini–Hochberg adjustment.

Statistically significant differences between the initial and final mean scores were observed for anxiety, depression, harm avoidance, and global psychological distress assessed using the VAS (all decreasing) and for the three maturity dimensions (all increasing) (**Table 3**). When considering the 25 facets, statistically significant differences between the initial and final mean scores were observed for Exploratory Excitability (NS1), Impulsiveness (NS2) (non-significant after the Benjamini–Hochberg procedure), Social Acceptance (C1), and all HA, RD, SD, and ST facets (**Table 1**).

**Table 4** shows statistically significant differences between the initial and final anxiety scores for all clinical motives underlying the request for psychotherapy. Group classification was based on patients’ presenting complaints rather than formal psychiatric diagnoses as defined by the ICD-10. Across the various groups, an overall increase was observed in SD scores—especially in the SD3 facet (Resourcefulness)—and a general decrease in HA, particularly in the HA2 facet (Fear of Uncertainty). In the grief/loss distress group and emotional disorder group, these changes were significant in paired t-tests but were not confirmed in ANCOVA models adjusted for age, sex, and baseline anxiety.

**Table 4:** Mean comparisons between initial and final TCI-125 dimension and facet scores according to the six different motives for requesting GT.

	Neurosis pathology (n=136)	Emotional disorders (n=86)	Self esteem pathology (n=41)	Psychosomatic disorder (n=12)	Grief/loss distress (n=29)	Others (n=15)
H/ANX	DEC****	DEC****	DEC****	DEC***	DEC****	DEC**
H/DEP	DEC****	DEC****	DEC**	–	DEC****	DEC***
NS	–	–	INC*^+^	–	–	–
NS1	INC*^+^	–	INC*^+^	–	–	–
NS2	–	–	–	–	–	–
NS3	–	–	–	–	–	–
NS4	–	–	–	–	–	
HA	DEC****	DEC****	DEC*^+^	–	DEC****	DEC*^+^
HA1	DEC***	DEC**	–	–	DEC*	DEC**
HA2	DEC**	DEC***	DEC*^+^	–	DEC*^+^	–
HA3	DEC****	DEC**	–	–	DEC*^+^	DEC*^+^
HA4	DEC*	DEC**	–	–	DEC**	–
RD	–	–	–	DEC*	–	–
RD1	–	DEC*^+^	–	DEC*^+^	–	–
RD3	INC****	INC*^+^	INC**	–	–	INC**^+^
RD4	DEC**	DEC*	DEC*^+^	–	DEC*	–
P	–	DEC**		–	–	–
SD	INC****	INC****	INC****	–	INC****	–
SD1	INC****	INC****	INC****	–	INC****	–
SD2	INC****	INC****	–	–	INC****	–
SD3	INC****	INC***	INC**	INC*^+^	INC**	–
SD4	INC**	INC***	–	–	INC***	–
SD5	INC***	INC****	–	–	INC**	–
C	–	–	–	–	INC**	–
C1	INC*	–	–	–	–	–
C2	–	–	–	–	–	–
C3	–	–	–	–	–	
C4	INC*	–	–	–	–	–
C5	–	–	–	–	–	–
ST	INC****	–	–	–	–	–
ST1	INC*	INC*	–	–	–	–
ST2	INC****	–	–	–	–	–
ST3	INC*^+^	–	–	–	INC*^+^	–

Significance was determined using paired *t*-tests. The ANCOVA-adjusted results by group are presented in the Results and Discussion sections. Benjamini–Hochberg procedure with *m* = 33 and *Q* = 5%.

H/ANX, HAD anxiety; H/DEP, HAD depression; NS, Novelty Seeking; HA, Harm Avoidance; RD, Reward Dependence; P, Persistence; SD, Self-Directedness; C, Cooperativeness; ST, Self-Transcendence; INC, Increase; DEC, Decrease.

^+^Non-significant after Benjamini–Hochberg adjustment.

****p ≤ 0.0001, ***p ≤ 0.001, **p ≤ 0.01, and *p ≤ 0.05.

With the VAS, statistically significant decreases in the measurements of distress were observed in both patients and therapists, with correlated scores at the initial and final sessions (r = 0.62, p < 0.001, and r = 0.73, p < 0.001, respectively).

## 4 Discussion

As assumed, the results of THEGETCI showed a significant increase in the three dimensions of character. As outlined in other studies, SD shows the greatest increase (46–48). Character is based on a continuous maturation process, and Self-Directedness increases throughout life (33, 49–51), confirming the hypothesis that maturity, an epigenetic factor, increases with experience. Each session, through the proposed techniques, is an experience that tends to develop the different facets of SD by reducing the patient’s repetitive behaviors, fostering awareness of “conservative adjustments”, and inviting the patient to adjust in new and creative ways to the therapeutic situation. This regularly renewed experience aims to develop maturity, autonomy, reliability, responsibility, awareness, and self-esteem. However, the evolution varied according to the identified problems. Interestingly, Cooperativeness increased less strongly, indicating a positive evolution in the ability to create constructive social relationships, particularly among those with better social acceptance. Overall, C remained broadly stable, except in the neurotic group, where social tolerance (Social Acceptance C1) and indulgence (Compassion C4) significantly increased. For this group, the data may indicate an improved capacity to build constructive relationships. The significant increase in Self-Transcendence (for its three facets—Self-Forgetfulness, Transpersonal Identification, and Spiritual Acceptance) is a corollary of the increase in maturity. During Gestalt therapy sessions, ST is stimulated by the quest for meaning in sensations, emotions, postures, and thoughts that emerge in the present situation. This results in a co-construction between patient and therapist that promotes detachment from oneself and recognition of shared human experience. A significant increase was observed for the Transpersonal Identification (ST2) facet. Meditative exercises can positively influence this aspect through the dual movement of relaxation and concentration they demand, promoting not only detachment from self and suffering but also focused attention on ongoing psychocorporeal manifestations. Spiritual Acceptance (ST3) can also be sharpened through the shared quest and co-construction of meaning, which can evolve into a possible spiritual and/or hermeneutic experience without falling into magical thinking or superstition.

The results also showed changes in temperament dimension scores, although to a lesser extent. HA was the only dimension that decreased significantly, as observed in other treatments [46–48]. This indicates that therapeutic work promotes increased energy, courage, and optimism in patients with depression or anxiety. This dimension aligns with the goals of accountability and patient empowerment in GT. All four facets—Anticipatory Worry (HA1), Fear of Uncertainty (HA2), Shyness with Strangers (HA3), and Fatigability and Asthenia (HA4)—decreased significantly, especially among patients with emotional, neurotic, and grief/loss problems.

The focus on the present—through meditation, breathing exercises, body relaxation, updating internalized parental or educational injunctions (introjects), and examining their effects on the therapeutic situation—can be assumed to influence the decrease in Anticipatory Worry (HA1) and Shyness with Strangers (HA3), particularly in emotional or neurotic problems (see **Table 2** for current strategies used in GT). Regarding loss and grief issues, dialogue, empathic listening to existential distress, feelings of abandonment or rejection, and developing past or anticipated relationships in role-playing tended to help patients recognize their own resources and reduce asthenia.

The reduction in HA was more discreet for self-esteem and psychosomatic problems, where doubt toward others and attachment to symptoms require more detailed psychotherapeutic work over a longer period due to alexithymia (52). In the emotional disorder and grief/loss groups, changes in SD and HA that were significant in paired t-tests did not remain significant after ANCOVA adjustment for age, sex, and baseline anxiety, suggesting that these effects may be explained by these covariates rather than by the treatment itself. It is also important to note that ANCOVA could not be applied in groups with small sample sizes (e.g., psychosomatic disorders and other problem groups), and due to limited statistical power in subgroup analyses, not all relevant covariates (e.g., education level, medication use, therapy duration, and depression) could be included in the adjusted models for every group. Notably, when baseline anxiety was excluded from the model in the emotional disorder group, the improvement in SD was significant again, suggesting that anxiety levels may mediate or obscure the treatment effect on this dimension in patients with emotional disorders.

While NS did not increase significantly overall, the NS1 facet (Exploratory Excitability) increased significantly in the neurotic and self-esteem groups (p < 0.05). This increase can be explained by accountability work in therapy, aimed at refocusing the patients’ demand for change toward themselves and their representations, rather than toward others or the therapist, in the form of victimization, claims, or grandiosity—common in self-esteem problems (53, 54). The stability of the other facets may indicate that impulsiveness, extravagance, and disorderliness (NS2, NS3, and NS4) are controlled in GT by a clear therapeutic framework, awareness of transference phenomena in the patient–therapist relationship, and exploration of their traumatic or phantasmatic origins.

The stability of RD may indicate the integrity and healthy narcissistic balance of Gestalt therapists, who aim primarily for patient autonomy while avoiding overly strong suggestions or manipulations. Nevertheless, there were differences between the facets of this dimension after treatment. The invitation to become aware of and share emotions can influence the Attachment (RD3) facet, which showed a significant increase in emotional, neurotic, and self-esteem pathologies. This seems to be a good indicator of the quality of the client–therapist bond, which was built and strengthened during sessions, but without fostering dependency (Dependence RD4), a facet that significantly decreased. Persistence remained stable because it is not a primary objective of GT, at least in its compulsive dimension. For all patients, the average HADS score at the beginning of treatment was approximately 17, corresponding to “severe anxiety or depression”, and decreased to approximately 12 post-treatment, corresponding to a “mild anxious or depressive state” (39). The decrease in anxiety was significant (p < 0.0001) for neurotic, emotional, grief/loss, and self-esteem problems, representing more than 90% of the patients (**Table 4**). This decrease remained significant, although more moderate, for psychosomatic problems (p = 0.003) and for other patients (p = 0.002). The reduction in depressive symptoms was also significant (p < 0.0001) for neurotic, emotional, and grief/loss problems and was more moderate for self-esteem issues (p < 0.003), while remaining stable for psychosomatic problems. In both cases, this represents a fundamental issue (55, 56) that may only evolve over a longer period through the exploration of trauma and its effects on the therapeutic relationship. Finally, the small patient sample for psychosomatic problems limits interpretation.

The VAS results, with blind assessments by both patients and practitioners, tended to show consistency in their perceptions of the positive evolution of the therapeutic process.

### 4.1 Limitations

This was a 1-year longitudinal study. However, it is not known whether this effect persists long after treatment. It would be interesting to investigate the long-term effects of GT and compare them with data from other established psychotherapies. Furthermore, we are not certain that patients who refused to participate in the study were actually identical to those included, nor whether they would have had a higher rate of failure than the study sample. We did not include a control group, which limited the scope of our study. However, we adjusted our results for many confounding factors such as age, sex, socioeconomic status, medication use, duration of therapy, and anxiety and depression scores. The therapist effect is indeed an important factor in any relational psychotherapy where the therapist is simultaneously one of the protagonists in the therapeutic relationship (57). In this study, all therapists had the same training, used the same method, and had at least 8 years of experience, which may have reduced differences between therapists’ results. However, future research should further explore the statistical and methodological considerations relevant to the therapist effect (58). Moreover, although there was a blind procedure for assessing effectiveness between the therapist and the patient and for recording initial assessments, self-assessment of effectiveness inevitably carries a bias of social desirability.

## 5 Conclusions

This study is one of the first to show the effectiveness of GT on personality dimensions through classical care. The rigorous evaluations presented herein demonstrate the importance of this technique. The specific contribution of GT lies in the therapist’s transmission of a particular attitude, consisting of a focus on the present, awareness, and responsibility: “These three elements (evaluation of current events, state of consciousness and responsibility) are the aspects of the same way of being in the world. Being responsible (able to respond) involves being present, being there. And being truly present is being conscious. And finally, being aware is presence (reality), a condition incompatible with the illusion of irresponsibility by which we avoid living our lives, rather than living them without caring what we think” (59).

Cloninger’s TCI-125 has shown its usefulness in assessing the effectiveness of GT. The results principally show that an increase in SD and a decrease in HA can serve as benchmarks for individual management. These significant personality changes can be considered indicators of progress in the psychotherapy process and, consequently, of the effectiveness of GT. Moreover, GT appears to improve state variables such as depression and anxiety, as well as subjective psychological distress. Recently, we developed a new version based on Cloninger’s biopsychosocial model, which integrates an eighth dimension for assessing alexithymia to better understand the evolution of patients with somatic disorders. This TCAI-150-LR (Temperament, Character, and Alexithymia Inventory with 150 items in a Lexical Revision) is currently under validation.

Further studies in other cohorts are needed to confirm the results presented here and to better determine the expected benefits for practitioners, as well as the patient profiles most likely to benefit from GT.

## Data Availability

The datasets presented in this study can be found in online repositories. The names of the repository/repositories and accession number(s) can be found below: https://doi.org/10.6084/m9.figshare.19345487.
