# Supplementary material for: Use of the Temperament and Character Inventory to describe the effectiveness of Gestalt therapy
Source: Front Psychiatry. 2025 Sep 26;16:1280954. doi: 10.3389/fpsyt.2025.1280954 (PMC12512043; doi:10.3389/fpsyt.2025.1280954)
Supplement: Supplementary file 1 [file DataSheet1.docx]

**List of members of THEGETCI Group:**

*the THEGETCI group (alphabetical):

Algaron Philippe (Paris), Aubry Murielle (Colmar), Barreau Françoise (Aubervilliers), Beauviala Muriel (Paris), Béranger Nelly (Saint-Nazaire), Berthelin Anne (Saint-Marcelin de Cray), Boulangé-Favaro Claire (Rennes), Brahim Roselyne (Le Perreux-sur-Marne), Butel Brigitte (Paris), Carpentier Anne (Paris), Charrier Carole (Rennes), Chatel Delphine (Rennes), Chauviré-Leduc Emmanuelle (Rennes), Chuche Ghislaine (Paris), Colavin-Gérard Magali (Nantes), Cornelis Marleen (Paris), Deblyck Nicolas (Nantes), Degas Thomas (Montreuil), Delahaie Gaëlle (Tours), Demaison Martine (Brive), Di Costanzo-Coucaud Nelly (Limoges), Domergue Luc (Rennes), Dorcet-Deschaumes Christine (Paris), Du-Jonchay Priscille (Nantes), Dumez Sylvie (Nantes), Dumez Laurent (Saint-Malo), Dupré Hélène (Larmor Baden), Duquenois Elise (Poitiers), Elhadad Anna (Bussy-Saint-Georges), Falgas Claude (Redon), Filippi Gaëlle (Paris), Fourrure Sophie (Paris), Gaille Olivier (Brest), Goudin Séverine (Vilennes-sur-Seine), Goutherot Céline (Quimper), Grosclaude Mireille (Rennes), Hérault Chrystèle (Plouër-sur-Rance), Julien-Siné Céline (Perpignan), Juston Didier (Bernay), Kervern Naïg (Rennes), Labro Anne-Marie (Lannion), Laroche Isabelle (Maule), Laurent Jean Marie (Blois), Lebas Cédric (Nantes) Lemaire Nicole (Les Clayes-sous-Bois), Lerasle Catherine (Londres), Lucas Aurélie (Saint-Malo), Masquelier Célestine (Versailles), Mauger-Combe Florence (Le Mans), Pasquet Laurence (Limoges), Pluvinage Séverine (Neuilly-sur-Marne), Raymondeau Christian (Limoges), Rodier Laurence (Saint-Jean d’Angely), Santoro-Kerviche Grazia (Paris), Sartori Anne (Rennes), Soulat Isabelle (Limoges), Turban Jean-Yves (Saint-Herblon), Vallejo Jean-Luc (Limoges), Winzenried Anne (Rennes).
